# Molecular magnetic resonance imaging of activated platelets allows noninvasive detection of early myocarditis in mice

**DOI:** 10.1038/s41598-020-70043-9

**Published:** 2020-08-06

**Authors:** Alexander Maier, Moritz Braig, Katharina Jakob, Thomas Bienert, Michaela Schäper, Annette Merkle, Carolin Wadle, Marius Menza, Irene Neudorfer, István Bojti, Peter Stachon, Daniel Duerschmied, Ingo Hilgendorf, Timo Heidt, Christoph Bode, Karlheinz Peter, Karin Klingel, Dominik von Elverfeldt, Constantin von zur Mühlen

**Affiliations:** 1 Department of Cardiology and Angiology I, Heart Center Freiburg University, Faculty of Medicine, University of Freiburg, Hugstetter Strasse 55, 79106 Freiburg, Germany; 2 Department of Radiology, Medical Physics, Medical Center - University of Freiburg, Faculty of Medicine, University of Freiburg, Freiburg, Germany; 3 Baker Heart & Diabetes Institute, Melbourne, Australia; 4 Cardiopathology, Institute for Pathology and Neuropathology, University Hospital Tuebingen, Tuebingen, Germany

**Keywords:** Cardiology, Medical research

## Abstract

MRI sensitivity for diagnosis and localization of early myocarditis is limited, although it is of central clinical interest. The aim of this project was to test a contrast agent targeting activated platelets consisting of microparticles of iron oxide (MPIO) conjugated to a single-chain antibody directed against ligand-induced binding sites (LIBS) of activated glycoprotein IIb/IIIa (= LIBS-MPIO). Myocarditis was induced by subcutaneous injection of an emulsion of porcine cardiac myosin and complete Freund’s adjuvant in mice. 3D 7 T in-vivo MRI showed focal signal effects in LIBS-MPIO injected mice 2 days after induction of myocarditis, whereas in control-MPIO injected mice no signal was detectable. Histology confirmed CD41-positive staining, indicating platelet involvement in myocarditis in mice as well as in human specimens with significantly higher LIBS-MPIO binding compared to control-MPIO in both species. Quantification of the myocardial MRI signal confirmed a signal decrease after LIBS-MPIO injection and significant less signal in comparison to control-MPIO injection. These data show, that platelets are involved in inflammation during the course of myocarditis in mice and humans. They can be imaged non-invasively with LIBS-MPIO by molecular MRI at an early time point of the inflammation in mice, which is a valuable approach for preclinical models and of interest for both diagnostic and prognostic purposes.

## Introduction

Besides myocardial ischemia, myocarditis is one of the most common causes of heart failure. The prevalence among young patients with sudden cardiac death is described within a range of 2–42%, and among patients with non-ischemic dilated cardiomyopathy within a range of 9–16%. The prognosis of progressing myocarditis is poor, symptoms are unspecific and diagnosis is challenging^1^.

Myocarditis is defined as an inflammatory process of the myocardium established by histological and immunohistochemical criteria associated with cardiac dysfunction. Several pathophysiological causes for myocarditis are known including infectious agents such as viruses, immune-mediated and toxic causes. Clinical symptoms, laboratory values, ECG and echocardiography are very often unspecific or inconclusive^1^.

To date, the diagnostic gold standard for diagnosis of myocarditis is endomyocardial biopsy (EMB). The Dallas criteria define myocarditis as histological evidence of inflammatory infiltrates within the myocardium associated with myocyte degeneration and necrosis of non-ischemic origin^2^. There are also immunohistochemical criteria, namely abnormal inflammatory infiltrate defined as ≥ 14 leucocytes/mm^2^, including up to 4 monocytes/mm^2^ with the presence of CD3 positive T-lymphocytes ≥ 7 cells/mm^2^^1^.

MRI is a promising non-invasive technique for diagnosis of myocarditis, especially in the context of improvements in scanners and sequences. Lake Louse Criteria have the largest evidence for diagnosis of acute myocardial inflammation by cardiac MRI. Current sequences are able to diagnose edema by T2-weighted images, hyperemia or capillary leak by early detection of gadolinium in T1-weighted images, late gadolinium enhancement as a sign of irreversible injury, ventricular dysfunction, wall thickness abnormalities, and pericardial effusion. T1 and T2 mapping techniques have increased sensitivity for pathological alterations^3^.

Despite significant improvements in the sensitivity of MRI sequences for inflammatory alterations, a lack still remains even with current standard of MRI and endomyocardial biopsy (EMB); for example in early stages of myocarditis, but also in convalescent and borderline myocarditis cases^3^. EMB is an invasive method, and inflamed regions cannot always be selectively reached by this procedure.

In a previous preclinical study we were able to image myocardial inflammation after ischemia/reperfusion injury with an MRI contrast agent targeting activated platelets^4^. This agent consists of an antibody against the ligand induced binding sites (LIBS) of activated platelets^5–8^ and microparticles of iron oxide (MPIO). The platelet specificity of this contrast agent was proven in several previous studies^9–13^. The LIBS epitope served as a target of platelet imaging also in PET^14^^,^ ultrasound^15–17^ and fluorescence computed tomography studies^18^. In this project, we hypothesized whether imaging of myocardial inflammation in a mouse model of myocarditis would be possible with the LIBS-MPIO contrast agent against activated platelets. However, platelet involvement in myocarditis was not described yet. It is well known that platelets play an important role in inflammatory and ischemic processes^19–21^. Hints for platelet involvement in myocarditis were given by another molecular imaging study, which used an ultrasound contrast agent against P-selectin^22^. P-Selectin is expressed on the vascular endothelium after inflammatory stimulation. Platelet surface receptors like GPIbα and PSGL-1 interact with endothelial P-Selectin and mediate platelet rolling. Firm adhesion is mediated via β_3_ integrins. Adherent platelets contribute to an inflammatory environment and recruit circulating leucocytes^19^.

Therefore, we here examine the role of platelets in a mouse model of myocarditis induced by porcine myosin, and performed molecular MRI with LIBS-MPIO to detect myocardial inflammation. To further confirm the translational potential of our findings in mice, we performed platelet staining in human endomyocardial biopsy specimens from patients with inflammatory cardiomyopathy.

## Methods

### Platelet specific contrast agent (LIBS-MPIO)

The monoclonal “anti-LIBS”-antibody binds to ligand induced binding sites (LIBS) on the glycoprotein IIb/IIIa receptor only in its active conformation^5^. The cloning, generation, and production of the anti-LIBS single-chain antibody have previously been described in detail^6^. To obtain a nonfunctional antibody for control purposes, exchange of the arginine in the RXD motif of the heavy chain CDR3 region of a single-chain antibody was performed. Generation and purification of the antibody were performed as described elsewhere^5,7,8^. For construction of the contrast agent, cobalt-functionalized autofluorescent microparticles of iron oxide (MPIOs) with a diameter of 1 μm were conjugated to the histidine tag of the anti-LIBS/control single-chain antibody as described in the manufacturer’s protocol (Dynal Biotech, Oslo, Norway) and in previously published studies^4,9–13^. Throughout this article, MPIOs conjugated to the anti-LIBS antibody are referred to as LIBS-MPIOs, and MPIOs conjugated to control antibody are called control-MPIOs. Each dose contained 4 × 10^8^ particles in 100 μl saline.

For finding the best time point for MR imaging of the inflamed myocardium, LIBS-MPIO was injected at day 2, 7, 14 and 21 after induction of myocarditis via the tail vein. Control-MPIO was injected the same way in different mice at this identified time point. Circulation of the contrast agent was allowed for 1 h. Thereafter, harvesting of the heart was performed.

For MRI, mice were positioned in the scanner, and contrast agent injection was performed via an 80-cm-long tail vein catheter. After slowly injection, flushing the tube with 100 μl saline ensured full injection.

### Myocarditis mouse model

Six to ten week old male BALB/c mice (Charles River, Sulzfeld, Germany) were subcutaneously injected with 150 μl of an emulsion of complete Freund’s adjuvant (CFA, Sigma-Aldrich, Taufkirchen, Germany) and porcine cardiac myosin (1 mg/ml, Sigma-Aldrich, Taufkirchen, Germany) in the neck on day-7 and 0. In a control group we injected an emulsion of incomplete Freund’s adjuvant (iCFA, Sigma-Aldrich, Taufkirchen, Germany) and porcine cardiac myosin^23,24^.

All experiments were conducted strictly according to the German animal protection law and in accordance with good animal practice as defined by the Federation of Laboratory Animal Science Associations (www.felasa.eu) and the national animal welfare body GV-SOLAS (www.gv-solas.de). The study was approved by the federal authorities in Freiburg and the Institutional Review Board (“Tierversuchskommission Regierungspräsidium Freiburg”) through animal experiment permission 35-9185.81/G-15/83.

### MRI protocols

All MR experiments were performed on a dedicated small-animal 7 T MRI system (BioSpec70/20, Bruker Germany) run with AVANCE III electronics, ParaVision 6 software together with a 2-channel cryogenically cooled mouse head surface coil.

The animals were placed head first in the supine position, and a reference probe consisting of 1% agarose in H_2_O (v/v) was attached to the coil for later signal quantification.

For animal monitoring and sequence triggering, neonatal ECG electrodes were attached to the front paws of the mice. In addition, a breathing sensor pad was placed underneath the animal, and the animal’s temperature was monitored and maintained at 37 °C by warm water–supported heating of the animal cradle. During MRI, anesthesia was maintained by slowly introducing Isoflurane up to a maximum of 2 Vol% in oxygen, stabilizing the animals at a breathing rate of ≈ 70 breaths per minute^4^.

Scouting scans were used to position the mouse heart in the center of the coil and for anatomical overview. A 3D Fast Low Angle SHot (FLASH) ECG gated sequence with dummy scans during respiration as described by Braig et al. was used to detect accumulated LIBS-MPIOs (echo time/repetition time, 3.5 ms/15 ms; flip angle 10°; bandwidth per pixel 1020 Hz)^25^. The gradient echo sequence with an echo time of 3.5 ms was chosen due to its sensitivity to induced T2* changes by the MPIOs and as a compromise between MPIO sensitivity and image quality (susceptibility artifacts).

Depending on the heartrate of the mouse around seven cine frames, covering systole and diastole were acquired with a temporal resolution of 15 ms. The chosen FoV (22 × 18 × 14 mm^3^) covered the heart from base to apex with a reconstructed isotropic resolution of (140 µm)^3^. The acquisition matrix was 80 × 64 × 50 corresponding to an acquired resolution of (280 µm)^3^. Because of the diffuse character of myocarditis with difficult prediction of its location and to avoid displacements of the ventricle in a 2D acquisition due to contrast bolus injections, a 3D volume was used for this study. Scan time was 15–20 min depending on the animal’s respiration.

After this initial scan the contrast agent was administered slowly. 12 mice were injected with LIBS-MPIO and 9 mice with control-MPIO. The first post contrast agent scan was obtained starting one minute after injection of the contrast agent with the same protocol as described above for the pre contrast agent scan. After the second scan, a third scan was performed and thereafter, mouse hearts were flushed with PBS, cut in two halves, and embedded in O.C.T.

### MRI data quantification

Bruker MR images were imported using an in-house coded MATLAB toolbox (MathWorks Inc, Natick, MA) with modified SPM8 (Statistical Parametric Mapping, Wellcome Centre for Human Neuroimaging, https://www.fil.ion.ucl.ac.uk/spm/) functions. For MRI signal quantification, 20 periodically chosen short axis slices of the left ventricle were circumscribed by manually surrounding the left ventricular epicardial and endocardial walls using a Matlab-based customized and modified software similar to the one described elsewhere^4^. This way, we selected 20 regions of interest (ROI_card_) of the left ventricular wall with a mean voxel count of 1,123 ± 31, corresponding to a volume of 157.1 ± 4.27 × 10^3^ µm^3^ per ROI_card_. Additionally, another 20 ROIs_ref_ (mean voxel count 58 ± 2 per ROI_ref_) corresponding to ROIs_card_ covered the voxels of the agarose reference. All MR images were normalized to the mean voxel intensity inside the reference ROI. After image normalization, the mean voxel intensity I_card_ of ROI_card_ before and after LIBS-MPIO administration was calculated. Pre contrast and post contrast voxel intensities were divided by the pre contrast voxel intensity mean to calculate spatial distributions of relative intensity changes compared to pre contrast values. Anatomical images displaying the relative intensity distribution changes were calculated by subtracting the normalized native images from the normalized images after LIBS-MPIO administration. The signal quantification strategy is also demonstrated by Fig. 5A.

### Platelet staining and MPIO quantification

Myocardial tissue was embedded with O.C.T., cut into 10-μm-thick sections, and fixed with acetone. For staining of platelets, a rat anti-mouse CD41 antibody was used (GTX 76011, GeneTex, Irvine, CA), and for control purposes, an IgG1 isotype control was used (MCA1211, Serotec, Puchheim, Germany). Secondary staining was performed with a biotinylated rabbit anti-rat IgG (BA-4001, Vector). Alkaline phosphatase and substrate kit (AK-5000 & SK-5100, Vector) and levamisole (X3021, DAKO) were used for detection. Finally, samples were embedded with Kaiser’s glycerol gelatin (Merck, Darmstadt, Germany) until adequate staining.

Platelets were counted in two representative pictures of × 20 magnification out of the inflamed myocardium of two different CD41–stained slices. For counting MPIOs, the inflamed myocardium of 10 representative CD41 stained slices was examined carefully.

### Human myocarditis specimens

Paraffin embedded 4 μm thick sections of human endomyocardial biopsies with inflammatory cardiomyopathy, which were not required any more for routine diagnosis underwent immunohistochemical CD41 staining and incubation with LIBS-MPIO. Informed consent was obtained from all subjects. The experiments were approved by the ethics committee, University Hospital Tuebingen (138/2004 V) and carried out in accordance with the guidelines of the Institute for Pathology and Neuropathology, University Hospital Tuebingen.

CD41 staining was performed with a rabbit monoclonal anti-CD41 antibody (1:200, ab134131, Abcam, Cambridge, UK). As detection system we used the VisUCyte HRP Polymer Mouse/Rabbit from R&D, Minneapolis followed by staining with HistoGreen (Linaris, Dossenheim) as substrate.

For the incubation of human myocarditis specimens with LIBS- or control-MPIO the contrast agent was diluted in PBS resulting in 4 × 10^8^ particles per 1 ml PBS. After deparaffinization one specimen was incubated with either the diluted LIBS- or control-MPIO contrast agent for 1 min each. Thereafter sections were washed with PBS three times for 5 min each. MPIOs were counted manually in 5 representative sectors of the specimen in × 100 magnification.

### Statistics

Statistical analysis was supported by GraphPad Prism (Version 5.0, GraphPad Software Inc.,). Results are given as dot-plot graphs with means. For statistical analysis of two groups, a Mann–Whitney test was performed. For analysis of data with > 2 groups, the Kruskal–Wallis test followed by a Dunn’s multiple comparison test was used. Comparisons of MRI signal intensities in the LIBS and control groups after MPIO application were undertaken with a 2-way multiple comparison ANOVA. Test results were considered significant at values of p < 0.05.

### Ethics approval and consent to participate

All experiments were conducted strictly according to the German animal protection law and in accordance with good animal practice as defined by the Federation of Laboratory Animal Science Associations (http://www.felasa.eu) and the national animal welfare body GV-SOLAS (http://www.gv-solas.de). The study was approved by the federal authorities in Freiburg and the Institutional Review Board through animal experiment permission 35-9185.81/G-15/83.

## Results

### Platelet infiltration and LIBS-MPIO binding is highest at early stages of myocarditis

**Figure 1:**
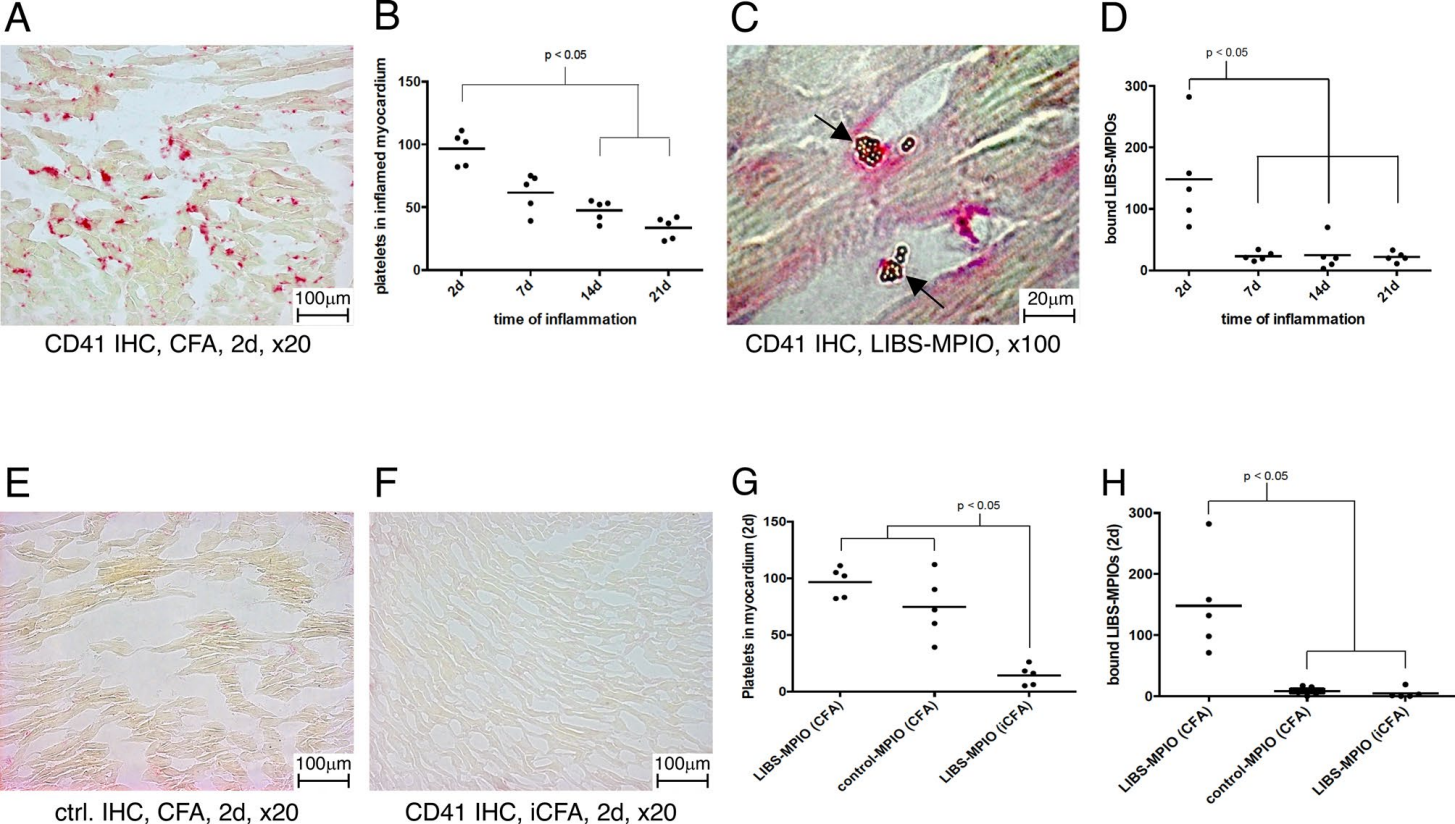
(**A**) CD41 platelet immunohistochemistry of murine myocarditis 2 days after induction of myocarditis (× 20). Platelets were stained red. (**B**) Histological platelet count showed significant higher platelet numbers at early time points after induction of myocarditis compared to later time points. (**C**) Round golden LIBS-MPIOs bind to platelets in inflamed myocardium. They are highlighted with arrows. (**D**) Quantification of bound LIBS-MPIOs in the myocardium showed significant higher binding 2 days after induction of myocarditis compared to later time points. (**E**) Control antibody staining of murine myocarditis induced with CFA and porcine cardiac myosin (× 20). (**F**) CD41 immunohistochemistry of iCFA and myosin injected mice 2 days after injection. No relevant platelet accumulation was observed. (**G**) iCFA and myosin injected mice developed significantly less platelets accumulation in the myocardium at 2 days after injection compared to CFA and myosin injected mice. (**H**) Both control-MPIO injected myocarditis mice and LIBS-MPIO injected sham mice (iCFA + myosin) had significantly less MPIO accumulation in the myocardium compared to LIBS-MPIO injected myocarditis mice (CFA + myosin).

Using CD41 staining in murine myocarditis we observed platelets within the myocardium (Fig. 1A). The number of platelets was highest at early stages of the inflammatory process. Two days after induction of myocarditis, the number of platelets was significantly higher compared to later time points at 14 and 21 days after the induction (p < 0.05, Fig. 1B). The activated platelet-specific contrast agent LIBS-MPIO bound specifically to platelets in the inflamed myocardium at early stages of myocarditis. A histological example of platelet-bound LIBS-MPIOs can be found in Fig. 1C. We observed most LIBS-MPIOs in the myocardium 2 days after induction of the myocarditis compared to later time points (7 d, 14 d, 21 d, p < 0.05, Fig. 1D). A negative control staining of inflamed myocardium from CFA injected mice with a control antibody is shown in Fig. 1E. Specificity of LIBS-MPIO binding was confirmed with two control groups. A CD41-stained example of iCFA and porcine cardiac myosin injected mice without relevant myocarditis can be found in Fig. 1F. The number of platelets was significantly lower in iCFA injected mice compared to CFA injected ones, as depicted in Fig. 1G (p < 0.05). No significant MPIO binding could be found after injection of control-MPIO and LIBS-MPIO injection to iCFA injected mice without relevant myocarditis (p < 0.05, Fig. 1H).

### Human specimens of inflammatory cardiomyopathy show CD41-positive areas

**Figure 2:**
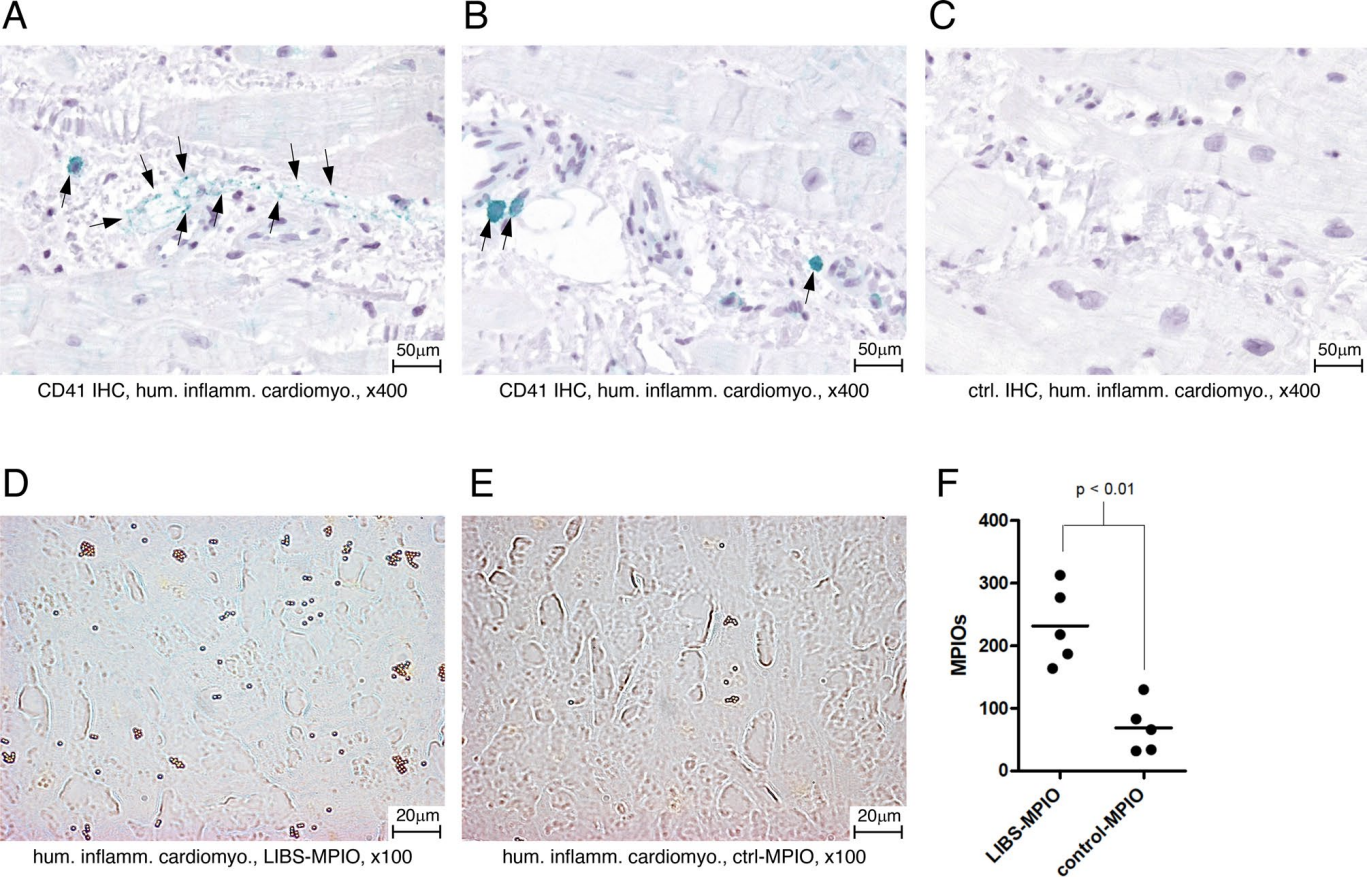
(**A**,**B**) CD41 immunohistochemistry of human inflammatory cardiomyopathy. The CD41 antigen is stained green. CD41 positive cells are highlighted with black arrows. (**C**) Control antibody staining of human inflammatory cardiomyopathy specimen. No CD41 positivity is seen (**D**) Human inflammatory cardiomyopathy specimen incubated with LIBS-MPIO. MPIOs can be identified as golden pellets. (**E**) Human inflammatory cardiomyopathy specimen incubated with control-MPIO. (**F**) Quantification of LIBS-MPIO binding to a human inflammatory cardiomyopathy specimen showed significantly more binding of LIBS-MPIO than control-MPIO.

We stained endomyocardial biopsy specimens from patients with inflammatory cardiomyopathy for CD41 antigen and found CD41 positive cells in the myocardium (Fig. 2A,B). A negative control staining is shown in Fig. 2C.

### LIBS-MPIO binds to human specimens of inflammatory cardiomyopathy

Human endomyocardial biopsy specimens of inflammatory cardiomyopathy were incubated with LIBS- or control-MPIO. Counting of iron oxide particles after incubation showed a significant higher number in the LIBS-MPIO incubated specimen compared to the control-MPIO incubated specimen as seen in Fig. 2D–F (p < 0.01).

### LIBS-MPIO allows imaging of early inflammation in myocarditis

**Figure 3:**
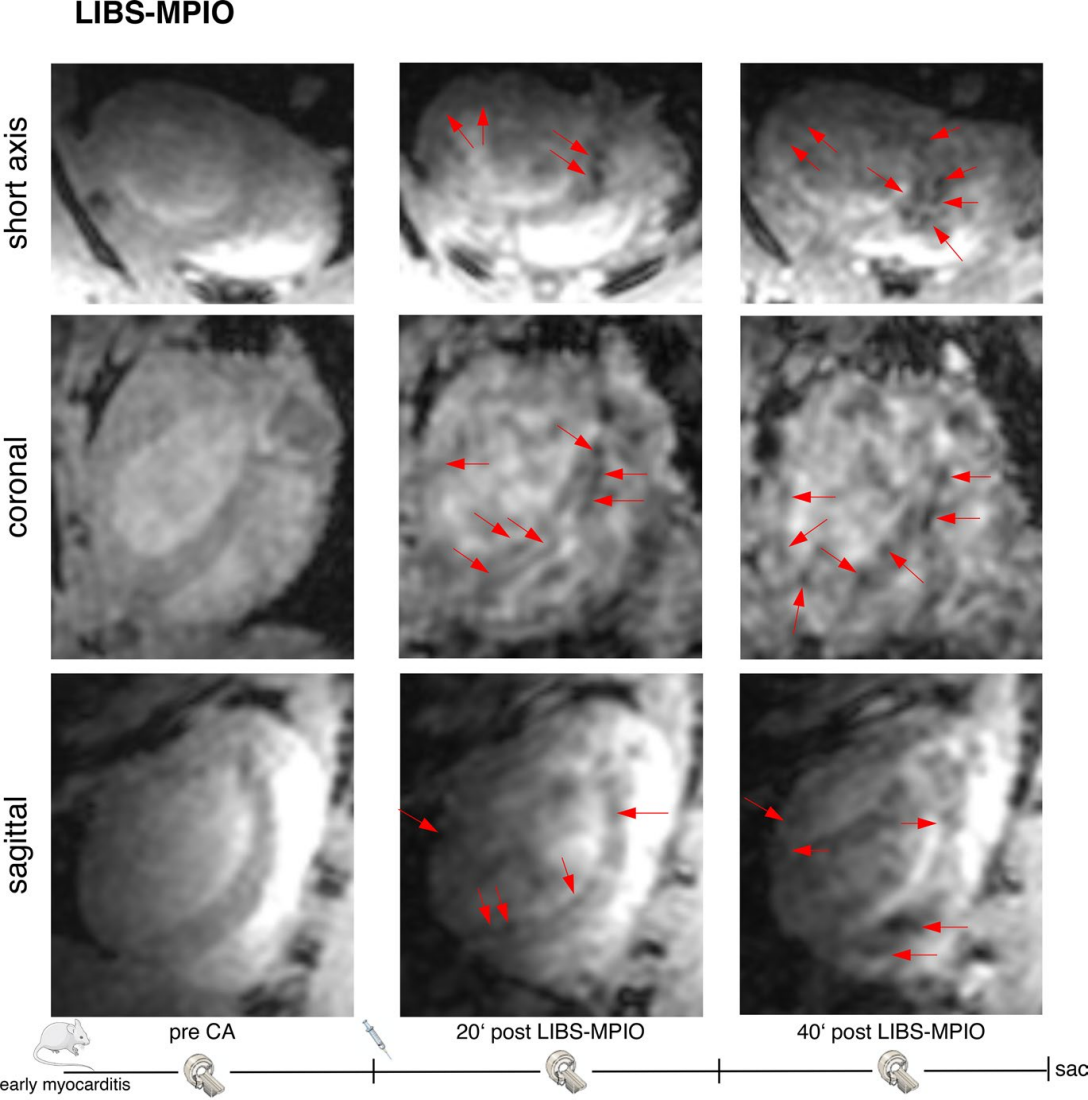
Enlarged in-vivo 3D MRI 2 days after myocarditis induction with LIBS-MPIO injection. The first picture of each row shows the pre contrast agent scan. The second and third pictures show the scans 20 and 40 min after LIBS-MPIO injection. The given minute numbers below the post injection images refer to the end of the scan. Red arrows highlight focal signal voids after LIBS-MPIO injection in short axis, coronal and sagittal slices. (Drawings are provided by Servier Medical Art by Servier, licensed under Creative Commons Attribution 3.0 Unported License).

**Figure 4:**
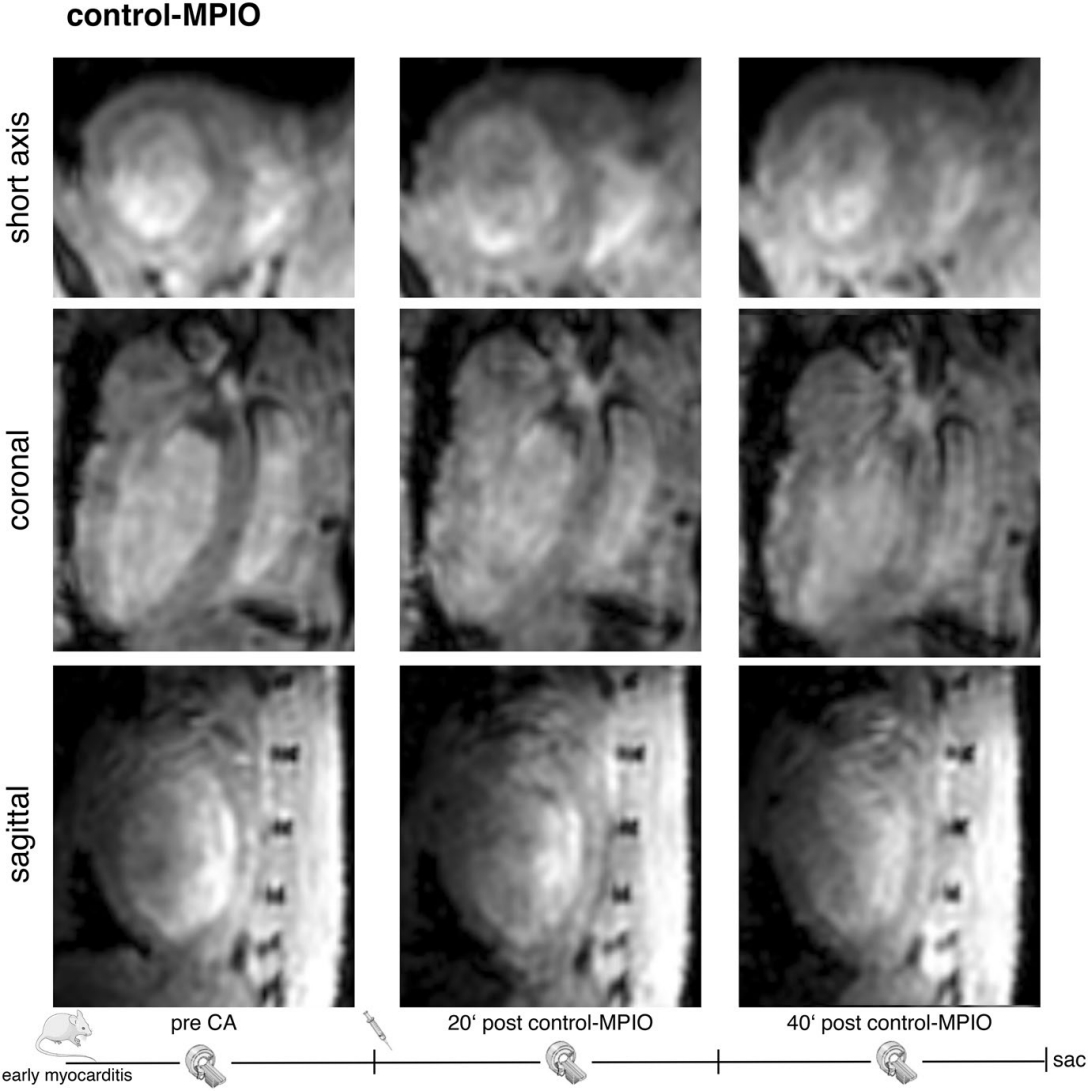
Enlarged in-vivo 3D MRI 2 days after induction of myocarditis with control-MPIO injection. The first picture of each row shows the pre-contrast agent scan. The second and third picture show scans 20 and 40 min after control-MPIO injection. The given minute numbers below the post injection images refer to the end of the scan. No relevant signal void visible after control-MPIO injection. (Drawings are provided by Servier Medical Art by Servier, licensed under Creative Commons Attribution 3.0 Unported License).

3D in-vivo MRI of mice with 2 days old myocarditis was performed before and after LIBS-MPIO or control-MPIO injection. Several spots of pronounced signal attenuation within the myocardium were visible after LIBS-MPIO injection as highlighted in Fig. 3 with red arrows in axial, coronal and sagittal slices. In contrast, after injection of control-MPIO no signal void was detectable within the myocardium as seen in Fig. 4.

### Signal quantification demonstrates myocardial signal decrease after LIBS-MPIO injection in early myocarditis

**Figure 5:**
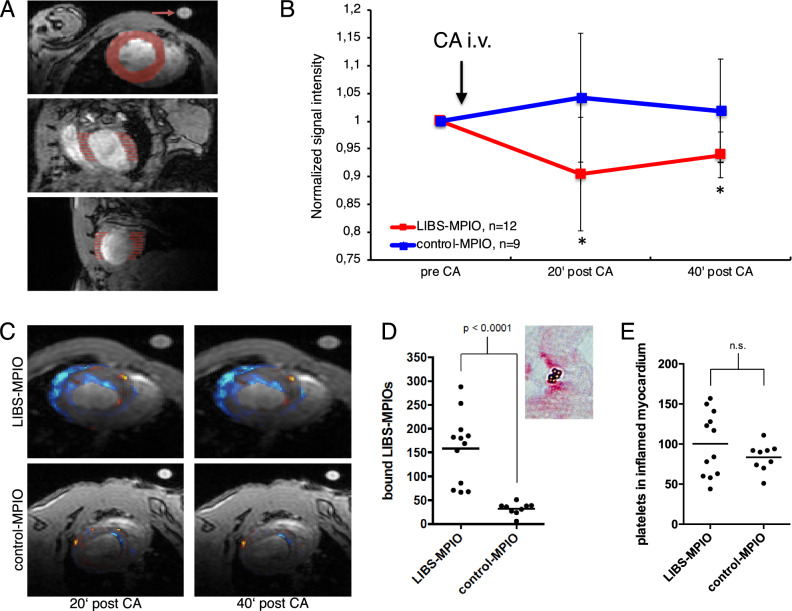
(**A**) Visualization of the signal quantification method. The signal in 20 periodically chosen slices of the myocardium was normalized to a 1% agarose reference probe and the pre-contrast agent scan. The reference probe is highlighted with a red arrow. (**B**) After injection of LIBS-MPIO a signal decrease was observed. The signal differed significantly from control-MPIO injected mice 20 and 40 min after injection. Animal numbers LIBS-MPIO: n = 12, control-MPIO: n = 9. (**C**) Visualization of the signal decrease in LIBS-MPIO injected mice (upper row) and control-MPIO injected mice (lower row). Blue indicates a lower signal in the myocardium compared to the pre contrast agent scan. (**D**) Histological counting of MPIOs in the myocardium showed significantly higher numbers of LIBS-MPIOs compared to control-MPIOs. (**E**) Platelet count was similar in both groups.

After normalization of the myocardial MRI signal to the agarose reference and pre contrast agent scans, a decrease of the myocardial MRI signal after LIBS-MPIO injection was observed, whereas no decrease was observed after control-MPIO injection. LIBS-MPIO injected mice showed a significantly lower MRI signal compared to control-MPIO injected mice (p < 0.05, Fig. 5B). In LIBS-MPIO injected mice the MRI signal dropped by 9.5 ± 10.2% 20 min after injection and 6.1 ± 4.1% 40 min after injection. Figure 5C shows a visualization of the relative signal decrease. Blue indicates a lower signal in the myocardium compared to the pre contrast agent scan.

Histological MPIO count within the myocardium showed a significantly higher number of platelet-bound MPIOs in LIBS-MPIO injected mice compared to control-MPIO injected mice (p < 0.0001, Fig. 5D). Platelet numbers in the myocardium showed no statistically significant difference between both groups (Fig. 5E).

## Discussion

Within this study, we were able to demonstrate that platelets play a time-dependent role in myocarditis in mice and are also involved in humans myocarditis. Thus, activated platelets may be used as a molecular MRI target to visualize early myocarditis with the activated platelet-specific contrast agent LIBS-MPIO.

Most LIBS-MPIOs bound to platelets at early time points of the inflammatory process, although platelets are also present at later time points. An explanation for this phenomenon could be the specificity of the LIBS antibody for activated platelets, which might play a major role in early stages of the inflammation. A second reason can be the higher number of platelets itself within the inflamed myocardium at early time points of the inflammatory process.

Early imaging of myocardial inflammation in myocarditis is of central clinical interest due to a lack of sensitivity of other noninvasive methods for diagnosis of early myocarditis. ECG, echocardiography and laboratory values are unspecific. Current human MRI sequences are able to detect (1) myocardial edema by T2 weighted imaging, T2 mapping, native T1 mapping and extracellular volume mapping, (2) hyperemia and capillary leakage by early gadolinium enhancement and native T1 mapping, (3) myocardial fibrosis and necrosis by late gadolinium enhancement and extracellular volume mapping^3^. By adding activated platelets to the already established imaging targets, the diagnostic range could be expanded by a potentially early occurring pathophysiological player. This preclinical study demonstrated, that LIBS-MPIO platelet imaging could provide early hints for the beginning of myocardial inflammation in mice. Clinically, this might guide early endomyocardial biopsy with subsequent early initiation of an anti-inflammatory therapy. Furthermore, the location of the endomyocardial biopsy could be selected more specifically to locate early inflammatory areas.

Other experimental approaches for imaging of myocarditis mainly used macrophages as the imaging target. This was possible with high sensitivity by fluorine MRI^26,27^ and with iron oxide particles^28^. These studies injected the contrast agent or tracer, respectively, 1 or 2 days prior imaging due to relatively slow uptake by the targeted macrophages. LIBS-MPIO platelet imaging did not need this long latency from injection to imaging in our study. The fast binding properties of the LIBS antibody enables LIBS-MPIO in this preclinical study as a specific tool for rapid assessment of inflammation in the myocardium. In a clinical scenario, this could lead to faster decisions towards endomyocardial biopsy or immunosuppressive treatment. Compared to macrophage imaging, LIBS-MPIO might be more suitable for early time points of the inflammation, when macrophages are not present in a high number.

Another interesting experimental myocarditis imaging approach by Helluy et al. focused on imaging of tissue damage by iron depositions with T2* MRI in a viral myocarditis model^29^. In-vivo imaging of cell damage causing iron deposition was possible 14 days after induction of myocarditis whereas our LIBS-MPIO approach can image platelets at a much earlier time point.

Two recent studies by Werner et al. showed the possibility of longitudinal imaging of macrophages in a rat model of autoimmune myocarditis with fluorodeoxyglucose (FDG) and 11C-Methionin by positron emission tomography (PET)^30,31^. Major limitations of PET imaging are its relatively long scan time and the patients’ exposure to radioactivity.

With the increased availability of combined PET/MR scanners, there is the clinically important opportunity to detect biopsy-negative myocarditis^32^. In patients with suspected myocarditis, FDG-PET was described in good agreement with MR findings^33^.

Due to biological safety aspects, we decided for an autoimmune model of myocarditis. This model is similar to the clinical more relevant viral model of myocarditis. Previous studies identified myosin as the major autoantigen in CVB3 induced autoimmune myocarditis^24^. Whether platelets are also involved in a viral model needs to be confirmed in future studies.

Revealing the mechanistic background of platelet involvement in myocarditis was not part of this study. The underlying mechanism of platelet involvement might be an activation of the endothelium by an immune reaction with subsequent activation of platelets by P-selectin. P-selectin is a cell adhesion molecule expressed on the surface of activated endothelium recruiting leukocytes and is also involved in platelet activation^34^. This potential mechanism was proposed by a study from Steinl et al., which successfully used P-selectin as a target epitope for imaging of inflammation in myocarditis^22^.

For the detected platelet involvement in the human biopsy specimens used in our study, an activation of platelets by the endomyocardial biopsy procedure itself cannot be ruled out.

The ability of LIBS-MPIO to discriminate between myocarditis and myocardial infarction is limited, since platelet activation occurs in both diseases within the myocardium. Thus, in a clinical scenario in which both myocardial infarction and myocarditis are suspected, coronary artery disease still needs to be ruled out. LIBS-MPIO might be helpful in such a scenario, since it has shown its ability to image thrombus formation in preclinical mouse models of acute coronary and carotid artery thrombosis by MRI^10,12^.

Correlation between MRI signal decrease and number of bound MPIOs in histology was not significant, although 20 slices for bound MPIOs in histology and the signal of 20 MRI slices were analyzed. Voxel size in MRI was limited to 140 µm^3^, whereas slice thickness in histology was 10 µm. Therefore, the analyzed regions are from similar anatomical positions but essentially not the same. Signal decrease in MRI may also occur from local field inhomogeneities and rapid MPIO turnover in the myocardium limiting the correlation of bound MPIOs and signal extinction. Cardiovascular MRI T2* mapping may be used to improve quantification of signal extinction but requires several echo times and therefore would have increased scan times beyond our limits. A correlation between severity of myocarditis and MRI signal was also not possible because of the early imaging and sacrificing time point of the mice. Relevant inflammatory infiltrates were not found at this early time point of the inflammation in HE staining.

A comparison between the occurrences of early edema by a T2 weighted imaging and early LIBS-MPIO accumulation was not part of this study. Therefore, a possible advantage of LIBS-MPIO over edema imaging in early detection of myocarditis could not be figured out. Unfortunately, we found no data about myocardial edema imaging and its occurrence in mice in the available literature.

Endomyocardial biopsy is usually taken from the right ventricle through a femoral or jugular vein access. In this study, we were not able to assess the free right ventricular wall by MRI due to its thin wall in mice. However, a signal void was visible in the septum, which is typically the area biopsies are taken and which could therefore serve as a target for LIBS-MPIO guided endomyocardial biopsy.

An important limitation of this study is the difficulty to directly apply the described contrast agent to patients. MPIOs are covered with polystyrene, which is potentially toxic in humans. We recently developed a potentially human-compatible iron oxide-based MRI contrast agent, which could overcome this limitation^35^. However, it was not the goal of the present study to use this potentially human-compatible contrast agent for a proof of concept. The anti-LIBS antibody is a single-chain antibody of small molecular weight (approximately 25 kDa), which has a low risk of causing immunoreactivity^36^. During all of the experiments performed for this and earlier studies, no animals showed signs of toxicity or symptoms of an allergic reaction.

Sensitivity of the contrast generated by targeted agents is an intrinsic limitation of MRI. A negative contrast agent generated by iron oxide demands a pre- and post-contrast comparison and requires increased scan times. However, the noninvasiveness and unnecessity of ionizing radiation of this imaging technique counterweights relative long scan times. Progress in research on the development of MRI contrast agents, MR scanners, and imaging sequences keeps promise for the clinical use of molecular MRI.

A possible translation into human application would have to take into account the larger size of the heart and the magnetic field strength. A field strength typically used in humans is 1.5 or 3 T. The reduced absolute image resolution should be overcompensated by the increase in object size, resulting in an improved image representation. For example, current scanners and sequences are able to detect ultra- small particles of iron oxide (USPIOs) in-vivo in humans after myocardial infarction at a field strength of 1.5 T^37^. Furthermore, in a previous in vitro MRI study, we have demonstrated the general feasibility of detecting LIBS-MPIOs at the clinically relevant field strength of 3 T^38^. However, the results of our study suggest that LIBS-MPIO could be a valuable approach for preclinical models.

## Conclusions

Platelets are involved in myocarditis-associated inflammation, as seen in murine autoimmune myocarditis as well as in human endomyocardial biopsy specimens of inflammatory cardiomyopathy. In mice, we found a time dependent involvement with higher platelet numbers in early stages of inflammation. At this stage, activated platelets can be imaged with LIBS-MPIO in molecular 3D in vivo-MRI in mice. In conclusion, activated platelets therefore represent a novel and early marker of myocarditis.

## Data Availability

All data generated or analyzed during this study are included in this published article.
